# Malignancy risk of hyperfunctioning thyroid nodules compared with non-toxic nodules: systematic review and a meta-analysis

**DOI:** 10.1186/s13044-021-00094-1

**Published:** 2021-02-25

**Authors:** Lorraine W. Lau, Sana Ghaznavi, Alexandra D. Frolkis, Alexandra Stephenson, Helen Lee Robertson, Doreen M. Rabi, Ralf Paschke

**Affiliations:** 1 Department of Medicine, Cumming School of Medicine, University of Calgary, Calgary, Canada; 2 Section of Endocrinology and Metabolism, Department of Medicine, Cumming School of Medicine, University of Calgary, Calgary, Canada; 3 Arnie Charbonneau Cancer Institute, Cumming School of Medicine, University of Calgary, Calgary, AB Canada; 4 Clinical Medicine. Health Sciences Library, University of Calgary, Calgary, Canada; 5 Department of Community Health Sciences, Cumming School of Medicine, University of Calgary, Calgary, AB Canada; 6 Departments of Oncology, Pathology, and Laboratory Medicine, Biochemistry and Molecular Biology, Cumming School of Medicine, University of Calgary, Calgary, Canada

**Keywords:** Thyroid nodules, Malignancy, Hot nodule, Thyrotoxicosis, Thyroid cancer

## Abstract

**Background:**

Hyperfunctioning or hot nodules are thought to be rarely malignant. As such, current guidelines recommend that hot nodules be excluded from further malignancy risk stratification. The objective of this systematic review and meta-analysis is to compare the malignancy risk in hot nodules and non-toxic nodules in observational studies.

**Methods:**

Ovid MEDLINE Daily and Ovid MEDLINE, EMBASE, Scopus, and Web of Science databases were searched. Observational studies which met all of the following were included: (1) use thyroid scintigraphy for nodule assessment, (2) inclusion of both hyperfunctioning and non-functioning nodules based on scintigraphy, (3) available postoperative histopathologic nodule results, (4) published up to November 12, 2020 in either English or French. The following data was extracted: malignancy outcomes include malignancy rate, mapping of the carcinoma within the hot nodule, inclusion of microcarcinomas, and presence of gene mutations.

**Results:**

Among the seven included studies, overall incidence of malignancy in all hot thyroid nodules ranged from 5 to 100% in comparison with non-toxic nodules, 3.8–46%. Odds of malignancy were also compared between hot and non-toxic thyroid nodules, separated into solitary nodules, multiple nodules and combination of the two. Pooled odds ratio (OR) of solitary thyroid nodules revealed a single hot nodule OR of 0.38 (95% confidence interval (CI) 0.25, 0.59), toxic multinodular goiter OR of 0.51 (95% CI 0.34, 0.75), and a combined hot nodule OR of 0.45 (95% CI 0.31, 0.65). The odds of malignancy are reduced by 55% in hot nodules; however, the incidence was not zero.

**Conclusions:**

Odds of malignancy of hot nodules is reduced compared with non-toxic nodules; however, the incidence of malignancy reported in hot nodules was higher than expected. These findings highlight the need for further studies into the malignancy risk of hot nodules.

**Supplementary Information:**

The online version contains supplementary material available at 10.1186/s13044-021-00094-1.

## Background

Autonomously hyperfunctioning thyroid nodules represent approximately 5–10% of all thyroid nodules. These so-called “hot nodules” are defined by increased radiotracer uptake compared to surrounding thyroid parenchyma on scintigraphy. Hot nodules can exist as a single hot nodule or as toxic multi-nodular goiters (TMNG). The degree of autonomous hyperfunction in hot nodules is variable, and some hot nodules may not produce sufficient levels of thyroid hormones to suppress TSH levels at initial presentation [1–4]. Clinical care pathways for the management of thyroid nodules recommend measurement of serum thyrotropin (TSH) followed by scintigraphy in patients with the presence of thyroid nodules and subnormal TSH levels [5]. Scintigraphy use in patients with normal TSH levels has been questioned [2] and is more commonly utilized in Europe [4].

Compared to non-toxic nodules, hot nodules are traditionally believed to have an exceptionally low rate of malignancy. This has led to widely-adopted recommendations by several guideline groups not to perform fine needle aspiration biopsy on these lesions irrespective of their size [1–7]. However, recent studies have challenged the presumed low-risk of malignancy in hot nodules, suggesting that the incidence of cancer has been underestimated [6–10]. In 22 patients who underwent thyroid surgery irrespective of functional nodule status, Ashcraft and Van Herle reported a malignancy risk of 4% in hot nodules [11, 12]. A recent study demonstrated higher than expected malignancy rates in hot nodules with an overall malignancy rate of 8.5% [13]. The reported malignancy rates of hot nodules ranges broadly from 0.34 to 44% among patients undergoing thyroid surgery [14, 15]. In comparison, the reported malignancy rate of non-toxic nodules ranges from 8 to 16% [7, 11–13, 16, 17].

Given the current recommendation against cytologic evaluation of hot nodules, and the widely variable malignancy rate reported in these lesions, there is a need to critically appraise the current literature in this area. Therefore, this systematic review aims to address the question: among those individuals undergoing thyroidectomy for benign indication, are hot nodules diagnosed by scintigraphy associated with a lower risk of thyroid malignancy compared with non-toxic thyroid nodules? Secondary objectives include comparison of malignancy risk in single compared with multiple hot nodules, assessment of reported carcinomas within compared to outside the hot nodule, association of hyperfunctioning on scintigraphy compared with biochemical hyperfunctioning (as determined by TSH levels) and its impact on malignancy, and the impact of inclusion of microcarcinomas on malignancy rates of hot nodules.

## Methods

### Protocol and registration

This systematic review was registered with a pre-published protocol on PROSPERO (CRD42019119204). Reporting was in accordance with the preferred reporting items for systematic review and meta-analyses (PRISMA) [18].

### Search strategy and databases

Two investigators (LL & RP) created a preliminary search strategy that was subsequently refined by a medical librarian (HLR). In brief, a search strategy aimed to include all articles from human studies published up to November 12, 2020 that utilized scintigraphy to assess functional status of thyroid nodules and subsequently included histopathologic data on these nodules. Complete search terms are available in Supplemental Fig. 1. Citations were found by searching the following databases from the first date available to November 12, 2020: Epub Ahead of Print, In-Process & Other Non-Indexed Citations, Ovid MEDLINE Daily and Ovid MEDLINE, EMBASE, Scopus, and Web of Science. Combinations of subject headings, keywords and synonyms used included all three key terms: 1) thyroid nodule, 2) hyperthyroidism, thyrotoxicosis, and hot nodule and 3) thyroid neoplasm, thyroid carcinoma, medullary carcinoma, follicular carcinoma, papillary carcinoma, and anaplastic carcinoma. The formalized search strategy is summarized in the Supplementary information.

### Study selection

After duplicates were removed, two reviewers (LL & AS) independently screened 1464 articles. Initial screen of the title & abstract for full text assessment was determined based on mention of thyroid nodule functional status and inclusion of surgical pathology. An additional 5 articles were added from other sources. These sources include review of references in published reviews and included articles, and additional articles recommended by expert researchers and clinicians in the field. Case reports, review articles, and small series (*n* ≤ 10) studies were excluded. Studies that included nodules noted outside the thyroid gland were also excluded. Inclusion criteria included studies that used thyroid scintigraphy (^131^I/^123^I or T^99m^) for nodule assessment, inclusion of both hyperfunctioning and normo−/hypo-functioning nodules based on scintigraphy, available postoperative histopathologic nodule results, and no age restriction. The reviewers (LL & AS) independently determined if studies met inclusion and exclusion criteria. Discrepancies were settled by a third reviewer (RP).

### Data extraction

Among the articles that met inclusion and exclusion criteria for analysis, the data extracted is summarized in Supplemental Table 1. In brief, quantitative measures included sample size, gender distribution, number of hot nodules and non-hot nodules and distribution of thyroid carcinomas. Binary measures included clear description of the thyroid carcinoma within the nodule, inclusion of microcarcinomas, and presence of genetic mutations. Data management was performed with Microsoft Excel. Furthermore, incidence of malignancy was calculated for all hot nodules and non-toxic nodules.

### Data analysis

Analyses were performed exploring the pooled odds ratio (OR) and 95% confidence interval (CI) of malignancy in: 1) single hot thyroid nodules compared with non-toxic nodules based on scintigraphy; 2) toxic multinodular goiters containing a hot nodule compared with non-toxic multinodular goiters; and 3) all hot nodules. Heterogeneity across studies was determined using Cochran’s Q and *I*^*2*^ statistic [19]. Due to the presence of significant heterogeneity, Mantel-Haenszel-weighted DerSimonian and Laird random-effects model were utilized [20]. Meta-regressions were not performed due to limited sample size. All analyses were performed using Stata 14.2 with an alpha of 0.1 and Review Manager 5.3 (Version 5.3.5, The Cochrane Collaboration, Copenhagen, Denmark).

### Quality assessment

The methodological assessment of included cohort studies was assessed by two independent reviewers (LL, RP) using the Newcastle-Ottawa Scale [21]. The role of this tool is to assess for patient selection bias, and for comparability of study groups and study outcomes.

## Results

### Search results

**Fig. 1:**
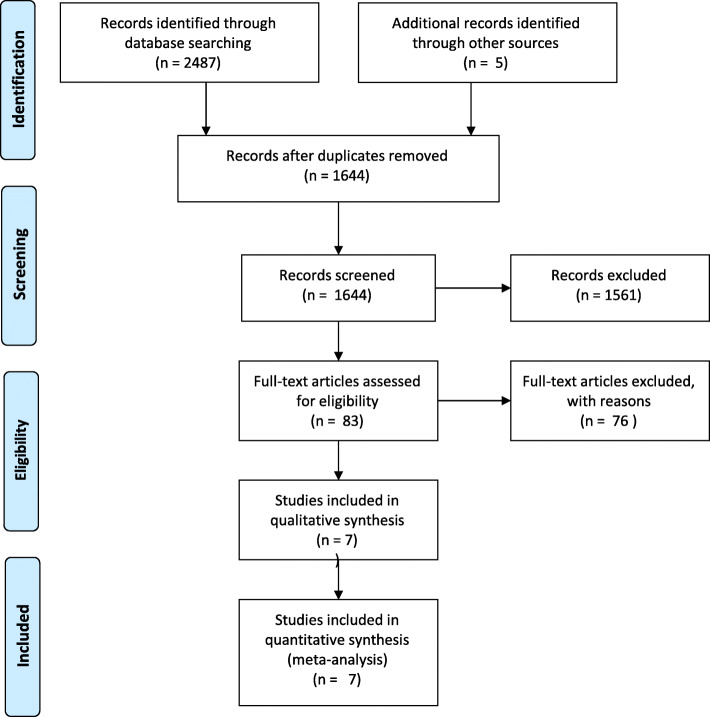
PRISMA Study flow diagram. Summary of the search strategy results based upon a pre-determined inclusion and exclusion criteria. Seven studies met criteria and their demographic data are summarized in Table 1

Our search results are summarized in Fig. 1. Among 2487 citations identified for review, there were 1644 remaining after removal of duplicates. Upon review of title and abstract, 83 full text articles were reviewed. Based on our exclusion criteria, 76 articles were excluded (reasons summarized in Supplementary Tables 2 and 3) with 7 studies included for qualitative and quantitative synthesis.

### Characteristics of included studies

A summary of the 7 observational studies included in our synthesis is presented in Table 1 [9, 22–27]. Publication dates ranged from 1994 to 2019. Studies originated predominantly from Europe, with 2 of 7 from Italy and 2 of 7 from Turkey. Total number of thyroid nodules across all studies was 7726, which ranged from 120 to 2870 nodules per study. Mean age ranged from 11.5 to 54 years old. Overall, most studies were surgical cohort studies that retrospectively examined predictors of malignancy. Thyroid carcinomas were diagnosed by fine needle aspiration biopsy (FNAB) and/or surgical histology. Scintigraphy was conducted with Tc^99m^ in 5 of 7 studies. In these five studies, scintigraphy was routinely performed in all the patient cohorts. Microcarcinomas were reported in 5 of 7 studies. Microcarcinomas comprised between 9.5 to 100% of the carcinomas reported in the studies. Among the 7 studies, only two study provided clear localization of the thyroid carcinoma within the hot nodule as these pediatric patients only had one nodule [23, 27]. In the other 4 studies, it is unclear if the carcinoma was confirmed within the hot nodule or in adjacent thyroid tissue.

**Table 1:** Characteristics of the 5 cohort studies included in qualitative and quantitative analysis

Authors	Year	Country	Sample Size	Mean Age	Retrospective/ Prospective	Diagnosis method	Radiotracer	Inclusion of microcarcinomas (%)	Carcinoma localized to hot nodule	Hot nodule – Hyperthyroid^**a**^ (%)	Hot nodule – Euthyroid^**a**^ (%)
Baser, H., et al. [22]	2019	Turkey	509	54.6	Retrospective	FNAB/Histology	Tc99m	Yes (?)	No	Unknown	Unknown
Corrias, A., et al. [23]	2010	Italy	120	11.5	Retrospective	FNAB(104) /Histology (63)	Tc99m	No	Yes	67	33
Derosa, G., et al. [24]	1994	Italy	619	46.0	Retrospective	FNAB/Histology	Tc99m	No	No	100	0
Dirikoc, A., et al. [25]	2017	Turkey	2870	49.0	Retrospective	Histology	Tc99m/131I	Yes (75.1%)	No	100	0
Mon, S. Y., et al. [9]	2018	USA	703	48.6	Prospective	FNAB	Iodine	Yes (9.5%)	No	67	33
Slijepcevic, N., et al. [26]	2015	Serbia	2466	54.0	Retrospective	Histology	Unknown	Yes (100%)	No	Unknown	Unknown
Tam, A., et al. [27]	2019	Turkey	439	46.0	Retrospective	FNAB/Histology	Tc99m	Yes (?)	Yes	Unknown	Unknown

^a^Hyperthyroid defined as suppressed TSH, and euthyroid defined as normal TSH

TSH level was measured in all studies. However, only two studies reported the TSH levels and correlated these levels with scintigraphy results [9, 23]. The three other studies did not directly report TSH levels for hot nodules [22, 24–27].

### Malignancy rate in hot nodules

**Fig. 2:**
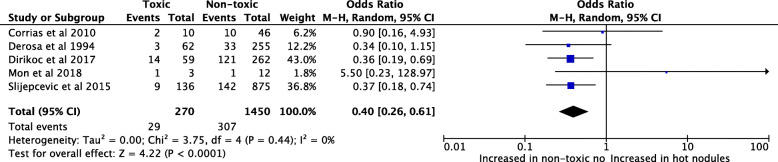
Pooled odd ratios for malignancy risk of single hot thyroid nodules compared with single non-toxic nodules based on scintigraphy

Hot nodules were differentiated into single hot nodules and TMNG. Similarly, non-toxic nodules were differentiated into single non-toxic nodules (NTN) and non-toxic multinodular goiters (MNG). Study outcomes for the odds ratio of single hot nodules versus single NTN are shown in Fig. 2. Pooled odds ratio in 5 studies involving 6778 nodules demonstrated a lower odd of malignancy in single hot nodules (OR = 0.38; 95% CI 0.25, 0.59; I^2^ = < 0.0002) compared to single NTN. Mon et al. represented a distinct outlier with an OR of 5.50 (95% CI 0.23, 128.97) [9]. Malignancy rates are reported malignancy per rates per nodule. Both Baser et al. and Tam et al. were excluded as it was unclear whether nodules were solitary or MNG [22, 27].

**Fig. 3:**
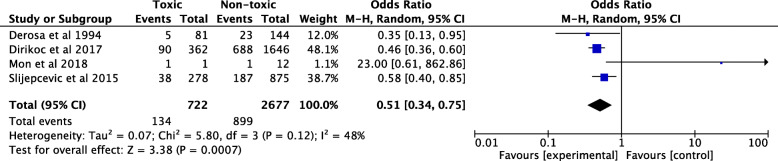
Pooled odds ratio for malignancy risk of toxic multinodular goiters (TMNG) containing a hot nodule compared with non-toxic multinodular goiters

The pooled odds ratio of 4 studies involving 6658 individuals demonstrated a lower odd of malignancy in TMNG compared to non-toxic MNG (OR = 0.51; 95% CI 0.34, 0.75; *I*^*2*^ = 48.3%). These outcomes are summarized in Fig. 3. Mon et al. was a clear outlier in comparison with the three other studies with an OR of 23 (95% CI = 0.61, 862.86) [9]. Corrias et al. was excluded from this analysis as MNGs were absent in their study [23].

**Fig. 4:**
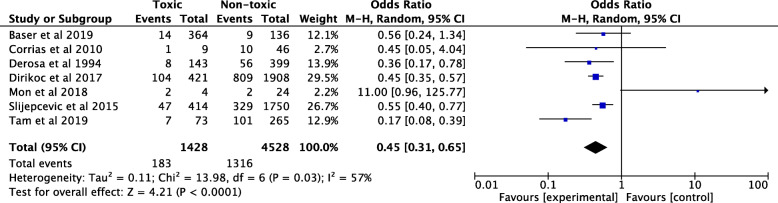
Pooled odds ratio for malignancy risk for all hot nodules including thyroid glands with single or multiple nodules

The overall pooled OR for all hot nodules, including both single and multiple nodules, was lower in comparison to all non-hot nodules (OR = 0.45; 95% CI 0.31, 0.65; *I*^*2*^ = 57%). These outcomes are summarized in Fig. 4.

Incidence of malignancy was calculated for all nodules and is summarized in Table 2. Among the 7 studies, the overall incidence of malignancy in all hot nodules ranged from 5 to 100% in comparison with non-toxic nodules, ranging from 3.8–46%. The FNA cytology and surgical histology results are also summarized in Table 2.

**Table 2:** Reported incidence of malignancy in all nodules including hot nodules and non-toxic nodules

Authors	Overall incidence of malignancy (%)	Overall proportion of hot nodules (%)	Incidence of malignancy in single hot nodules (%)	Incidence of malignancy in TMNG (%)	Incidence of malignancy in NTN (%)	Incidence of malignancy in MNG (%)	FNA Biopsy results	Malignant Surgical Histology
Baser, H., et al. (2019) [22]	5	73	N/A	N/A	3.8	6.6	80 – Nondiagnostic 259 – Benign 17 – AUS/FLUS 2 – FN/SFN 2 – SM 4 - Malignant	8 – PTC 2 – FTC 3 – TTUMP 1 - UTC
Corrias, A., et al. (2010) [23]	30	16	11	6	22	16	3 suspicious	1 - PTC (All 3 suspicious FNAB were benign)
Derosa, G., et al. (1994) [24]	10	26	5	25	13	42	Not reported	8 - PTC
Dirikoc, A., et al. (2017) [25]	34	34	24	8	46	14	^a^50 – Nondiagnostic 83 – indeterminate 13 – malignant	118 PTC 7 FTC 6 Other
Mon, S. Y., et al. (2018) [9]	13	12	33	100	8	8	2 - Benign 1 - AUS/FLUS	2 FTC (Both were FNAB benign)
Slijepcevic, N., et al. (2015) [26]	16	6	7	14	16	21	Not reported	9 PTC
Tam, A., et al. (2019) [27]	25	17	N/A	N/A	10	28	8 – Nondiagnostic 51 – Benign 3 – AUS/FLUS 2 – FN/SFN 1 - Malignant	10 PTC 4 FTC (7 malignancies located outside hot nodule)

*TMNG* toxic multinodular goiter, *NTN* non toxic nodule, *MNG* multinodular goiter, *FNA* fine needle aspirate, *AUS/FLUS* atypia of undetermined significance/follicular lesion of unknown significance, *SM* suspicious for malignancy, *PTC* papillary thyroid cancer, *FTC* follicular thyroid cancer, *TTUMP* thyroid tumour of unknown malignant potential, *UTC* undifferentiated thyroid cancer, Other includes Hurthle cell, medullary or undifferentiated

^a^Denotes cytology findings in all hyperthyroid patients

### Assessment of bias and quality of evidence

Risk of bias was assessed using the Newcastle-Ottawa assessment scale for cohort studies, which evaluated the quality of the evidence based on selection, comparability, and outcome (Table 3) [21]. Only one study was assessed as low risk with 6 stars; however, this study evaluated only pediatric patients [23]. All other studies were assessed as having high risk of bias as they were all surgical cohorts without a non-surgical (ie. medically managed) cohort for comparison, thus awarded 5 stars or less. Furthermore, Mon et al. was assessed with high risk of bias in comparability as this study selected specifically for patients with TSH receptor mutation without a mutation negative study control. Follow up duration and adequacy were not applicable to the assessment.

**Table 3:**
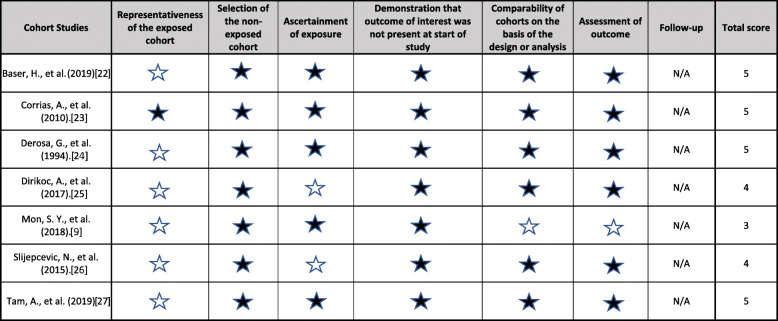
Summary of risk of bias assessment based on Newcastle-Ottawa Quality Assessment for Cohort Studies. A filled star denotes that a star has been awarded and that a study has been graded high quality. A blank star denotes that no star has been awarded and that the study has been graded as poor quality in that category. Total score indicates the total number of stars awarded in all categories. N/A denotes not applicable

### Post-hoc assessment of malignancy outcomes in studies reporting hot nodules only

Given the higher than expected incidence of malignancy in the included studies, studies that were excluded due to lack of non-toxic nodules were re-examined. Specifically, the incidence of malignancy in hot nodules was evaluated in single hot nodules and TMNG. These findings are reported in Supplementary Table 4. Quantitative assessment was not performed as the comparability of the studies was not appropriate. Incidence of malignancy ranged from 0 to 44% in single hot nodules, 0–26% in TMNG, and 0–29% in all hot nodules (single hot nodules and TMNG).

Furthermore, post hoc analysis of odds of malignancy in only adult patients without *a prior* knowledge of TSHR mutations is summarized in Supplemental Fig. 1. Pooled ORs of all hot nodules was lower than all non-toxic nodules (ORs 0.43, 95% CI 0.32, 0.58, *I*^*2*^ = 46%).

### Case reports identified through the search strategy

Based on our search strategy, 62 case reports of thyroid carcinoma within a hot nodule were identified with publication dates from 1972 to present. Demographic information was extracted from these case reports and are seen in Table 4. Patient age varied from 2 months to 74 years of age. Most hot nodules were single hot nodules, though some TMNGs were also included. Papillary thyroid carcinomas (PTCs) and follicular thyroid carcinomas (FTCs) were the most commonly reported malignancies. However, follicular variant of papillary thyroid carcinomas (fvPTC), Hurthle cell, anaplastic (with a concomitant hot nodule) and medullary thyroid carcinomas (described as a cold area of the HN) were also identified [28, 29].

**Table 4:** Summary of 62 case reports identified through our search strategy that reported thyroid carcinomas within hot nodules. (*AFTN* autonomously functioning thyroid nodules, *TMNG* toxic multinodular goiter, *FTC* follicular thyroid carcinoma, *FVPTC* follicular variant of papillary thyroid carcinoma, *MTC* medullary thyroid carcinoma, *PTC* papillary thyroid carcinoma)

Authors	Year	Study Title	Journal Title	Sample size	Age	Single or multiple toxic nodules	Type of thyroid carcinoma
Abs, R., et al.	1991	Hyperfunctioning metastatic follicular thyroid-carcinoma in Pendreds syndrome	Cancer	1	66	TMNG	FTC (metastatic)
Alaoui, N. I. and N. Ben Rais	2011	Association of hyperthyroidism and well-differentiated thyroid carcinoma (medullary excluded). A propos of seven cases.	Medecine Nucleaire-Imagerie Fonctionnelle Et Metabolique	7	41.4	4 Graves’ disease, 2 single AFTN, 1 TMNG	5 PTC, 2 FTC
Appetecchia, M. and M. Ducci [41]	1998	Hyperfunctioning differentiated thyroid carcinoma.	Journal of Endocrinological Investigation	1		AFTN	PTC
Ardito, G., et al. [42]	1997	Papillary thyroid carcinoma mimicking an autonomous functioning nodule.	European Journal of Surgical Oncology	1		AFTN	PTC
Bajja, M. Y., et al. [43]	2017	Mucinous carcinoma of the thyroid: A case report and review of the literature.	Annales D Endocrinologie	1	74	AFTN	Mucinous carcinoma of the thyroid
Barrande, G., et al.	1997	Two thyroid carcinomas mimicking toxic adenomas.	Presse Medicale	2	35 and 55	AFTN	
Becker, F. O., et al.	1963	The occurrence of carcinoma in “hot” thyroid nodules. Report of two cases.	Annals of Internal Medicine	2			
Bircan, R., et al.	2007	The second follicular thyroid carcinoma presenting as a hot thyroid nodule with a somatic 1486F TSH-Receptor (TSHR) gene mutation.	Hormone Research	1			FTC
Bitterman, A., et al. [44]	2006	Thyroid carcinoma presenting as a hot nodule.	Otolaryngology - Head & Neck Surgery	1	66, 57, 59	AFTN & TMNG	PTC & 2 FTC
Bommireddipalli, S., et al. [45]	2010	Follicular variant of papillary thyroid carcinoma presenting as a toxic nodule by I-123 scintigraphy.	Clinical Nuclear Medicine	1	63	AFTN	FVPTC
Bourasseau, I., et al.	2000	No evidence of thyrotropin receptor and G(s alpha) gene mutation in high iodine uptake thyroid carcinoma.	Thyroid	4	Mean age 41.1	3 AFTN, 1 TMNG	2 PTC and 2 FTC
Calimon, M. A. P. and S. W. Lim-Uy [46]	2014	Papillary Thyroid Carcinoma in an Autonomous Hyperfunctioning Thyroid Nodule.	Endocrine Reviews	1		AFTN	PTC
Camacho, P., et al. [47]	2000	A Phe 486 thyrotropin receptor mutation in an autonomously functioning follicular carcinoma that was causing hyperthyroidism.	Thyroid	1	49	AFTN	FTC
Campenni, A., et al. [32]	2011	Follicular variant of papillary thyroid carcinoma presenting as a toxic nodule in an adolescent girl	European Journal of Nuclear Medicine and Molecular Imaging	1	15	AFTN	PTC
Castelli, V., et al.	1994	Occurrence of papillary carcinoma in a hyperfunctioning thyroid nodule: report of a case and diagnostic considerations	Thyroidology	1			PTC
Cirillo, R. L., Jr., et al. [48]	1998	Metastatic pure papillary thyroid carcinoma presenting as a toxic hot nodule.	Clinical Nuclear Medicine	1		AFTN	PTC (metastatic)
Clement, K., et al. [49]	1991	Thyroid cancer revealed by an extinctive hot nodule	Presse Medicale	1	62		
Damle, N., et al. [33]	2011	Papillary carcinoma masquerading as clinically toxic adenoma in very young children.	Journal of Pediatric Endocrinology & Metabolism	2	6 months and 5y	AFTN	PTC
De Rosa, G., et al. [50]	1990	Thyroid carcinoma mimicking a toxic adenoma.	European Journal of Nuclear Medicine			AFTN	
Ducci, M., et al. [34]	1996	Differentiated carcinoma in autonomously functioning thyroid nodule: case report.	Acta otorhinolaryngologica Italica: organo ufficiale della Società italiana di otorinolaringologia e chirurgia cervico-facciale	1	13	AFTN	PTC
Einert, A., et al. [51]	1995	A combination of unifocal thyroid autonomy and follicular carcinoma - A case report	Radiologe	1	53	AFTN	FTC
Emmrich, P., et al. [52]	2001	Unifocal autonomous thyroid nodule and carcinoma.	Zentralblatt Fur Chirurgie	2		AFTN	FTC and PTC
Foppiani, L., et al. [53]	2005	Heterogeneous malignancy in toxic thyroid nodules	Journal of Endocrinological Investigation	3	68, 38, & 62	2 AFTN, 1 TMNG	3 FTC
Fuhrer, D., et al. [54]	2003	Two somatic TSH receptor mutations in a patient with toxic metastasising follicular thyroid carcinoma and non-functional lung metastases.	Endocrine-Related Cancer	1	59	TMNG	FTC
Fujimoto, Y., et al.	1972	Occurrence of papillary carcinoma in hyperfunctioning thyroid nodule. Report of a case.	Endocrinologia Japonica	1			
Fukata, S., et al. [55]	1987	Thyroid carcinoma and hot nodule	European Journal of Nuclear Medicine	1	70	AFTN	PTC
Gardner, D. and S. C. Ho	2014	A rare cause of hyperthyroidism: functioning thyroid metastases	BMJ Case Reports	1	66	TMNG	FVPTC
Gozu, H. et al. [67]	2004	Does a Leu 512 Arg thyrotropin receptor mutation cause an autonomously functioning papillary carcinoma?	Thyroid	1	?	TMNG	PTC
Kim, T. S., et al.	2007	A rare case of hyperfunctioning papillary carcinoma of the thyroid gland	Acta Oto-Laryngologica	1	32	AFTN	FVPTC
Kuan, Y. C. and F. H. Tan	2014	Thyroid papillary carcinoma in a ‘hot’ thyroid nodule.	Qjm	1	60	AFTN	FVPTC
Lado-Abeal J. et al [68]	2010	Identification of a paired box gene 8-peroxisome proliferator-activated receptor gamma (PAX8-PPAR gamma) rearrangement mosaicism in a patient with an autonomous functioning follicular thyroid carcinoma bearing an activating mutation in the TSH receptor	Endocrine-Related Cancer	1	55	TMNG	FTC
Lima, M. J., et al.	2018	Autonomously hyperfunctioning cystic nodule harbouring thyroid carcinoma - Case report and literature review.”	International Journal of Surgery Case Reports	1	49	AFTN	FVPTC
Majima, T., et al. [56]	2005	Papillary thyroid carcinoma without metastases manifesting as an autonomously functioning thyroid nodule.	Endocrine Journal	1	59	AFTN	PTC
Marcelino, M., et al. [28]	2014	Anaplastic carcinoma and toxic multinodular goiter: an unusual presentation	European Thyroid Journal	1	70	TMNG	Anaplastic thyroid carcinoma
Mircescu, H., et al. [35]	2000	Hyperfunctioning malignant thyroid nodule in an 11-year-old girl: pathologic and molecular studies.	Journal of Pediatrics	1	11	AFTN	PTC
Mirfakhraee, S., et al. [8]	2013	A solitary hyperfunctioning thyroid nodule harboring thyroid carcinoma: review of the literature	Thyroid research	1	29	AFTN	FTC
Nagai, G. R., et al.	1987	Scintigraphic hot nodules and thyroid carcinoma	Clinical Nuclear Medicine	3			2 FTC and 1 PTC
Nemec, J., et al. [57]	1980	Metastatic thyroid cancer with severe hyperthyroidism mimicking independent hyperfunctioning thyroid adenoma, showing transition to water-clear-tumour.	Endokrinologie	1		AFTN	FTC (metastatic)
Niepomniszcze, H., et al. [58]	2006	Follicular carcinoma presenting as autonomous functioning thyroid nodule and containing an activating mutation of the TSH receptor (T620I) and a mutation of the Ki-RAS (G12C) genes.	Thyroid	1	64	AFTN	FTC
Nishida, A. T., et al. [59]	2008	Multifocal hyperfunctioning thyroid carcinoma without metastases.	Auris, Nasus, Larynx	1	62	TMNG	PTC
Polyzos, S. A. and D. G. Goulis [30]	2011	Coincidental thyroid papillary microcarcinoma in a patient treated for a toxic adenoma of the thyroid.	Archives of Iranian Medicine	1		AFTN	PTC (microcarcinoma)
Rees, D. O., et al. [36]	2015	Follicular variant of papillary thyroid carcinoma: an unusual cause of thyrotoxicosis.	BMJ Case Reports	1	16	AFTN	FVPTC
Rivas, I., et al. [29]	1995	Medullary thyroid carcinoma mimicking an autonomous functioning nodule	Journal of Endocrinological Investigation	1	42	AFTN	MTC
Rubenfeld, S. and T. M. Wheeler	1988	Thyroid cancer presenting as a hot thyroid nodule: report of a case and review of the literature	Thyroidology	1		AFTN	
Ruggeri, R. M., et al. [37]	2013	Follicular variant of papillary thyroid carcinoma presenting as toxic nodule in an adolescent: coexistent polymorphism of the TSHR and Gsalpha genes.	Thyroid	1	15	AFTN	FVPTC
Russo, D. et al. [70]	1997	Detection of an activating mutation of the thyrotropin receptor in a case of an autonomously hyperfunctioning thyroid insular carcinoma	JCEM	1	60	AFTN	Insular carcinoma
Russo, D. et al. [69]	1999	A Val 677 activating mutation of the thyrotropin receptor in a Hurthle cell thyroid carcinoma associated with thyrotoxicosis	Thyroid	1	42	AFTN	Hurthle cell carcinoma
Sablayrolles, B., et al.	1983	Thyroid carcinoma presenting as a hot nodule in a child	Archives Francaises De Pediatrie	1			
Salih, A. M., et al.	2016	Hyperfunctioning papillary thyroid carcinoma: A case report with literature review.	nternational Journal of Surgery Case Reports	1	40	TMNG	PTC
Sandler, M. P., et al. [60]	1988	Thyroid carcinoma masquerading as a solitary benign hyperfunctioning nodule	Clinical Nuclear Medicine	1		AFTN	PTC
Sato, Y., et al.	1998	Hyperfunctioning thyroid adenoma concomitant with papillary thyroid carcinoma, follicular thyroid adenoma and primary hyperparathyroidism.	Endocrine Journal	1	67	TMNG	PTC
Schmidt, S., et al.	2016	Hyperfunctioning thyroid nodules (toxic adenoma)-underestimated risk of malignancy?	Langenbeck’s Archives of Surgery	1	72	TMNG	PTC
Schneider, P. W., et al. [61]	2000	A clear cell variant of follicular carcinoma presenting as an autonomously functioning thyroid nodule.	Thyroid	1		AFTN	FTC
Sevinc, B., et al.	2018	Papillary thyroid carcinoma after radioactive iodine treatment for toxic thyroid nodule: Case report.	Marmara Medical Journal	1	55	AFTN	PTC
Siddiqui, A. R. and S. Karanauskas [38]	1995	Hurthle cell-carcinoma in an autonomous thyroid nodule in an adolescent	Pediatric Radiology	1	16	AFTN	Hurthle cell carcinoma
Simsek, E., et al.	2014	Metastatic Papillary Thyroid Carcinoma in an Autonomous Hyperfunctioning Thyroid Nodule in an Adolescent	Endocrine Reviews	1		AFTN	PTC (metastatic)
Sobel, R. J., et al.	1985	Papillary carcinoma and the solitary autonomously functioning nodule of the thyroid.	Israel Journal of Medical Sciences	3		AFTN	PTC
Stahl, A., et al. [62]	2002	Differentiated thyroid carcinoma in a scintigraphically hot nodule: diagnosis and interdisciplinary therapeutical approach.	Wiener Klinische Wochenschrift	1	57	AFTN	Metastatic differentiated thyroid carcinoma
Tangari, A., et al. [31]	2011	Hot nodule harboring a papillary microcarcinoma in a girl from an iodine sufficient area.	Hormone Research in Paediatrics	1	3	AFTN	PTC (microcarcinoma)
Tfayli, H. M., et al. [39]	2010	Papillary thyroid carcinoma in an autonomous hyperfunctioning thyroid nodule: case report and review of the literature	Thyroid	1	11	AFTN	PTC
Uludag, M., et al.	2008	Autonomously functioning thyroid nodule treated with radioactive iodine and later diagnosed as papillary thyroid cancer.	Hormones	1	36	AFTN	PTC (columnar type)
Wong, C. P., et al. [63]	2003	Thyrotoxicosis: a rare presenting symptom of Hurthle cell carcinoma of the thyroid.	Clinical Nuclear Medicine	1		AFTN	Hurthle cell carcinoma

Among these 62 case reports, only two (4%) reported microcarcinomas within the hot nodules [30, 31]. In all 9 pediatric studies, there was sufficient evidence to support the presence of the thyroid carcinoma within the hot nodule [31–39]. In the 53 adult studies, 49% of studies had sufficient evidence to demonstrate thyroid carcinoma presence within the hot nodule [8, 29, 35, 40–63].

## Discussion

This systematic review and meta-analysis of observational studies comparing the malignancy rate of hot nodules compared with non-toxic thyroid nodules demonstrated a reduced malignancy rate in hot nodules; however, the rate was not as low as previously expected. Therefore, the findings of this review prompt us to question the widely adopted recommendation to avoid cytologic evaluation of hot nodules, based on the belief that hot nodules harbour a significantly lower malignancy rate than non-toxic nodules. Our findings cannot definitively support or refute this recommendation; however, this review gives us important insight into the methodological and evidence limitations in this area of the literature, including the need for meticulous cytologic-histologic and imaging correlation of nodules, and the need to explicitly report malignancy rates with and without inclusion of incidental papillary microcarcinomas. Each of these issues will be discussed in detail below.

### Location of the thyroid carcinoma within the hot nodule

A major challenge in the assessment of thyroid malignancy, particularly in multinodular goiters, is the location of the malignancy. It is not uncommon for a malignant nodule to co-exist with a benign nodule within the same thyroid lobe. This challenge can also be applied to hot nodules. Schroder and Marthaler evaluated 63 publications describing the presence of hot nodules with concurrent follicular or papillary thyroid cancer [64]. Out of the 63 publications, only 10 provided unequivocal confirmation of the carcinoma within the hot nodules, whereas in the other studies, it was uncertain whether the malignancy was found within the hot nodule or an adjacent non-toxic nodule. Interestingly, this study together with Pazaitou-Panaylotou et al described increased mortality in patients with carcinomas detected within the hot nodule [64, 65].

The identification of the carcinoma within the hot nodule can be technically difficult and requires close interdisciplinary collaboration. Localisation of the thyroid carcinoma in a specific nodule is particularly difficult in multi-nodular thyroid glands. However, accurate cytologic-histologic correlation of carcinomas is critical to understanding the true malignant potential of hot nodules [65]. Among the five studies included in this systematic review, Corrias et al identified the location of the carcinoma [23]. This study differed from the other four studies in that only pediatric patients were included. Given the increased malignancy risk reported in pediatric thyroid nodules compared to the adult population, malignancy rates found in pediatric populations cannot be extrapolated to the adult population [66]. In all 9 pediatric case reports there was sufficient evidence to support the presence of the thyroid carcinoma within the hot nodule as there was a single hot nodule being investigated, which correlated to location of the carcinoma on pathology [31–39]. In the 53 adult studies, nearly half (49%) of the studies demonstrated sufficient evidence of the thyroid carcinoma within the hot nodule based on the presence of a single hot nodule on scintigraphy with the location of the carcinoma on pathology [8, 29, 35, 40–63, 67–70]. The other case reports were confounded by the presence of multiple thyroid nodules, and did not clearly delineate the location of the hot nodule on scintigraphy with pathology.

### Inclusion of microcarcinomas

The percentage of carcinomas that were microcarcinomas in the seven included studies ranged from 9.5 to 100%. The increased detection of papillary thyroid microcarcinomas (defined as tumours less than or equal to 10 mm) has contributed significantly to the rise in incidence of thyroid cancer over the last few decades [71]. Microcarcinomas can be found in up to 35% of post-mortem studies [72]; most of these lesions are believed to be clinically insignificant. This has led to the current American Thyroid Association (ATA) recommendation to monitor sonographically suspicious or biopsy-proven papillary microcarcinomas, in an effort to prevent over-diagnosis and over-treatment of asymptomatic disease. In future studies, these low-risk microcarcinomas should either be analyzed separately, or excluded from the analysis of malignancy rate, to reflect the true risk of clinically significant malignancy in the study population.

### Mon et al. as the study outlier

The use of molecular diagnostics is gaining increasing recognition in the assessment of indeterminate thyroid nodules [73]. A clear outlier in this review is a study that deliberately selected indeterminate nodules with TSHR mutations identified by molecular diagnostic testing of indeterminate thyroid nodules [9]. Among the 16 TSHR mutation positive patients with available histology, 3 patients had evidence of thyroid cancer. This study represents a highly selected group with an unusual way of diagnosing hot nodules that is very distinct from the other study populations. A major deficiency in this study is the lack of appropriate clinical diagnosis of hot nodules prior to FNA and molecular diagnostics. TSH was only measured in 27 of the 703 thyroid samples tested for mutations and rearrangements and scintigraphy was used in only 4 of the 6 patients with suppressed TSH. Thus, the OR for this group cannot be generalized for hot nodules.

### Limitations

The notion that hot nodules rarely harbour malignancy is based on studies conducted in the 1960s to 1980s that examined scintigraphy in an undifferentiated patient population with thyroid nodules [12, 74]. At that time, the prevalence of thyroid nodules was estimated at 4 to 7% in the general adult population, with the risk of malignancy ranging from 10 to 20% [75, 76]. Since then, the prevalence of thyroid nodules has increased to 19–67% of the adult population based on increased use of and advances in ultrasonography, with similar malignancy rates of 8–16% [17, 77].

A wide variation of incidence rate of malignancy was reported in both hot nodules and non-toxic nodules in our study. A major confounder in all studies was the inclusion of only patients undergoing partial or total thyroidectomy. Given that these patients were selected for thyroidectomy instead of treatment with antithyroid medication or radioactive iodine therapy, there exists the potential for a selection bias influencing our primary outcome of the true rate of malignancy in hot nodules. For example, in the cohort of patients selected for thyroidectomy, as opposed to monitoring or radioactive iodine therapy, one reason for surgical intervention could be a high-risk sonographic pattern in the index hot nodule or other concurrent non-index lesions. In this cohort, it would be logical to see a higher rate of malignancy than expected. Furthermore, rate of malignancy may also vary based on geographical location, and local clinical practices (predominance of surgical resection versus treatment with radioactive iodine).

### Summary

Current guidelines for the differential diagnosis and treatment of thyroid nodules recommend clinical assessment and measurement of serum TSH levels [5, 78]. In patients with low TSH levels, the next recommendation involves thyroid scintigraphy with further malignancy risk stratification applied only to non-toxic nodules. The AACE/AME guideline recognise that in geographic regions with past or present iodine deficiency scintigraphy is used as part of the evaluation of patients with MNG and that TSH may remain unsuppressed even when autonomy is present [4].

Based on this systematic review, we were unable to identify a prospective study that directly compared the malignancy risk of hot nodules with non-toxic nodules in adults. Also, each included study contained one or more limitations that negatively impacted its ability to answer our primary question (see Table 1). The lack of a well conducted prospective study assessing the malignancy risk in all patients with hot nodules, together with the identification of 62 case reports identifying thyroid carcinomas within hot nodules, challenges the hypothesis that hot nodules are rarely malignant.

With limitations in mind, this systematic review demonstrates that the odds of malignancy in hot nodules are reduced by 49–62% compared to non-toxic nodules. However, the overall rate of malignancy observed in hot nodules is higher than expected. Traditionally, hot thyroid nodules were thought to rarely harbour malignancy with rates reported as low as 0.34% [14]. Higher incidence of malignancy in hot nodules was observed in the seven included studies ranging from 10 to 34% (Table 1) [9, 23–26]. FNA biopsy results available for 4 studies demonstrate a low diagnostic yield of FNA cytology for the diagnosis of malignancy (Table 2). A large number of studies were excluded from analysis for the inclusion of only hot nodules without a comparison with non-toxic nodules (Supplemental Table 3) [7, 10, 13–15, 65, 72, 79–98]. Furthermore, the search strategy identified 62 case reports that described the presence of thyroid malignancy within a hot nodule (Table 4).

In summary, this systematic review highlights the need for further research into the malignancy risk assessment of hot nodules. There is sufficient evidence to question the notion that hot nodules rarely harbour thyroid cancer. To adequately address this question, a study of adult patients would need to incorporate both scintigraphically hot and non-toxic nodules, resected for any indication, with histologic correlation of the location of the nodule by pre-operative imaging (ultrasound and scintigraphy) and histologic examination, and exclusion of low-risk papillary microcarcinomas. Furthermore, if hot nodules were to be subjected to further assessment, the ultrasonographic malignancy risk stratification would need to be assessed for this specific population.

## Supplementary Information

**Additional file 1: Supplemental Table 1.** Summary of data points extracted. **Supplemental Table 2.** Summary of excluded full text articles. **Supplemental Table 3.** Reasons for article exclusion. **Supplemental Table 4.** Incidence of malignancy in hot nodules reported in studies examining only hot nodules. **Supplemental Figure 1.** Pooled odds ratio of combined hot nodules compared with non-toxic nodules excluding pediatric patients and those with known TSHR mutations.

## Data Availability

All data generated or analysed during this study are included in this published article and its supplementary information files.

## Abbreviations

TMNG: Toxic multinodular goiters
TSH: Thyrotropin/Thyroid-stimulating hormone
PRISMA: Preferred reporting items for systematic review and meta-analyses
FNAB: Fine needle aspiration biopsy
NTN: Non-toxic nodules
MNG: Multinodular goiters
PTC: Papillary thyroid carcinomas
fvPTC: Follicular variant of papillary thyroid carcinomas
ATA: American Thyroid Association
AACE/ACE/AME: Amercian Association of Clinical Endocrinologist (AACE), American College of Endocrinology (ACE) and Associazione Medici Endocrinologi (AME)
