# Supplementary material for: Malignancy risk of hyperfunctioning thyroid nodules compared with non-toxic nodules: systematic review and a meta-analysis
Source: Thyroid Res. 2021 Feb 25;14:3. doi: 10.1186/s13044-021-00094-1 (PMC7905613; doi:10.1186/s13044-021-00094-1)
Supplement: Supplementary file 1 — Additional file 1: Supplemental Table 1. Summary of data points extracted. Supplemental Table 2. Summary of excluded full text articles. Supplemental Table 3. Reasons for article exclusion. Supplemental Table 4. Incidence of malignancy in hot nodules reported in studies examining only hot nodules. Supplemental Figure 1. Pooled odds ratio of combined hot nodules compared with non-toxic nodules excluding pediatric patients and those with known TSHR mutations. [file 13044_2021_94_MOESM1_ESM.docx]

**SUPPLEMENTAL INFORMATION**

Study Protocol

FOCUSED QUESTION:

Among those individuals undergoing thyroidectomy for a benign indication, does pre-surgical thyroid hyperfunction based on scintigraphy, relative to non-toxic nodules decrease the risk of thyroid malignancy?

Inclusion criteria:

- Original articles that used thyroid scintigraphy (with either iodine 123 or technetium-99m) for nodule assessment
- Available postoperative histopathologic diagnosis of nodules
- No date restriction
- Include all ages
- English language studies

Exclusion criteria:

- Case reports, animal studies and reviews
- Excluded if nodules were taken outside of the thyroid gland

Search strategy:

Citations were found by searching the following databases from the first date available to May 23, 2018: Epub Ahead of Print, In-Process & Other Non-Indexed Citations, Ovid MEDLINE Daily and Ovid MEDLINE, EMBASE, Scopus, and Web of Science. Combinations of subject headings, keywords and synonyms used included all three key terms: 1) thyroid nodule, 2) hyperthyroidism, thyrotoxicosis, and hot nodule and 3) thyroid neoplasm, thyroid carcinoma, medullary carcinoma, follicular carcinoma, papillary carcinoma, and anaplastic carcinoma.

Database: Ovid MEDLINE(R) Epub Ahead of Print, In-Process & Other Non-Indexed Citations and Ovid MEDLINE(R) Daily and Ovid MEDLINE(R) <1946 to May 22, 2018> (Updated on November 12, 2020

Search Strategy:

--------------------------------------------------------------------------------

1 Thyroid Nodule/ (5002)

2 (Thyroid adj3 Nodule*).mp. (9573)

3 1 or 2 (9573)

4 Hyperthyroidism/ (25538)

5 hyperthyroid*.mp. (34631)

6 hyper-thyroid*.mp. (73)

7 Thyrotoxicosis/ (3888)

8 thyrotoxicosis.mp. (8398)

9 (hyperfunction* adj3 nodule*).mp. (212)

10 (hyper-function* adj3 nodule*).mp. (6)

11 (autonomous* function* adj3 nodule*).mp. (253)

12 (hot adj3 nodule*).mp. (424)

13 (toxic adj3 nodule*).mp. (263)

14 or/4-13 (38726)

15 Thyroid Neoplasms/ (45452)

16 (thyroid adj3 (neoplasm* or cancer* or carcinoma* or tumour* or tumor*)).mp. (56873)

17 (thyroid adj3 adenoma*).mp. (2341)

18 ((medullary or follicular or papillary or anaplastic or hurthle) adj2 (neoplasm* or cancer* or carcinoma* or tumour* or tumor* or adenoma*)).mp. (44731)

19 or/15-18 (74523)

20 3 and 14 and 19 (629)

21 exp animals/ not (exp animals/ and humans/) (4463904)

22 20 not 21 (624)

23 limit 22 to english language (501)

**Supplemental Table 1: Summary of data points extracted**

| **Quantitative Measures** | **Binary Measures** |
| --- | --- |
| Sample size | Clear description of methods to identify the thyroid carcinoma in the hot nodule itself |
| Gender distribution | Retrospective versus Prospective |
| Age with standard deviation | Diagnosis method (FNAB vs histology) |
| Total number of AFTN  Total number of TMNG  Combined number of AFTN & TMNG | Microcarcinomas (<1cm) vs Macrocarcinomas (≥1cm) |
| Total number of NTA  Total number of NTMNG  Combined number of NTA & NTMNG | Presence of TSHR mutation |
| Malignancy rate of AFTN  Malignancy rate of TMNG  Combined malignancy rate of AFTN & TMNG | Presence of GSalpha mutation |
| Malignancy rate of NTA  Malignancy rate of NTMNG  Combined malignancy rate of NTA & NTMNG | Presence of other common mutations |
| Percentage of papillary thyroid cancers |  |
| Percentage of follicular thyroid cancers |  |
| Percentage of undifferentiated thyroid cancers |  |

Abbreviations: AFTN – autonomously functioning thyroid nodules; TMNG – toxic multinodular goiter; NTA – non-toxic thyroid adenoma, NTMNG – non-toxic multinodular goiter, FNAB – fine needle aspirate biopsy, TSHR – Thyroid stimulating hormone receptor

**Supplemental Table 2: Summary of excluded full text articles**

| **Reasons for exclusion** | **#** |
| --- | --- |
| 1) Absence of scintigraphy | 20 |
| 2) Absence of histological information | 9 |
| 3) Conference abstract | 3 |
| 4) Non-English full text | 9 |
| 5) Absence of hot nodules | 2 |
| 6) Absence of non toxic nodules | 33 |
| **Summary of exclusions** | **76** |

**Supplemental Table 3: Reasons for article exclusion**

| Authors | Absence of scintigraphy | Absence of histological information | Conference abstract | Non-English Full Text | Absence of non-toxic nodules | Absence of hot nodules |
| --- | --- | --- | --- | --- | --- | --- |
| Adam, B., et al. (2017). |  |  |  |  | Yes |  |
| Adas, M., et al. (2015). |  | Yes |  |  |  |  |
| Ahuja, S. and H. Ernst (1991). |  |  |  |  | Yes |  |
| Alonso, O., et al. (1996). | Yes |  |  |  |  |  |
| Als, C., et al. (1997). |  |  |  | Yes |  |  |
| Als, C., et al. (2002). |  |  |  |  | Yes |  |
| Angusti, T., et al. (2000) |  |  |  |  | Yes |  |
| Arai, M., et al. (1990). | Yes |  |  |  |  |  |
| Arturi, F., et al. (1998). | Yes |  |  |  |  |  |
| Badellino, F., et al. (1991). | Yes |  |  |  |  |  |
| Balasubramaniam, S., et al. (2012). | Yes |  |  |  |  |  |
| Bari, AKMF. et al. (2017) |  | Yes |  |  |  |  |
| Barry, M. C., et al. (1996). |  | Yes |  |  |  |  |
| Bayo, J. A. H., et al. (2010). |  |  | Yes |  |  |  |
| Balazs, G., et al. (1979). |  |  |  |  |  | Yes |
| Berker, D., et al. (2011). |  |  |  |  | Yes |  |
| Blum, M., et al. (1975). | Yes |  |  |  |  |  |
| Boi, F., et al. (2000). | Yes |  |  |  |  |  |
| Boiadzhieva, P. and G. Stoianova (1983). |  | Yes |  |  |  |  |
| Boostrom, S. and M. L. Richards (2007). | Yes |  |  |  |  |  |
| Botrugno, I., et al. (2011). | Yes |  |  |  |  |  |
| Bourasseau, I., et al. (2000). |  | Yes |  |  |  |  |
| Britvin, T., et al. (2011). |  |  | Yes |  |  |  |
| Brkljacic, B., et al. (2001). |  | Yes |  |  |  |  |
| Calkovsky, V. and A. Hajtman (2009). | Yes |  |  |  |  |  |
| Cakir, M., et al. (2007). |  |  |  |  | Yes |  |
| Campenni, A., et al. (2017). |  |  |  |  |  | Yes |
| Cappelli, C., et al. (2006). |  |  |  |  | Yes |  |
| Cassol, C. A., et al. (2010). | Yes |  |  |  |  |  |
| Celano, M., et al. (2003). |  | Yes |  |  |  |  |
| Chigot, J. P., et al. (2000). |  |  |  | Yes |  |  |
| Choong, K. C. and C. R. McHenry (2015). |  |  |  |  | Yes |  |
| Christensen, S. B., et al. (1984). |  |  |  |  | Yes |  |
| Croom, R. D., 3rd, et al. (1987). | Yes |  |  |  |  |  |
| Daali, M. and T. Tajedine (2003). |  |  |  | Yes |  |  |
| Das, A. B., et al. (1996). |  |  |  |  | Yes |  |
| Daumerie, C., et al. (1998). |  |  |  | Yes |  |  |
| David, E., et al. (1995). |  |  |  |  | Yes |  |
| de Luca, F., et al. (1986). |  |  |  |  | Yes |  |
| Derrien, C., et al. (2001). | Yes |  |  |  |  |  |
| Diaconescu, M. R., et al. (2007) | Yes |  |  |  |  |  |
| Dirikoc, A., et al. (2015). |  |  |  |  | Yes |  |
| Ducassou, D., et al. (1975). |  |  |  | Yes |  |  |
| Erdogan, M. F., et al. (2003). |  |  |  |  | Yes |  |
| Eszlinger, M., et al. (2014). |  |  |  |  | Yes |  |
| Gabriele, R., et al. (2003). |  |  |  |  | Yes |  |
| Giles, Y., et al. (2008). |  |  |  |  | Yes |  |
| Gulcelik, M. A., et al. (2006). | Yes |  |  |  |  |  |
| Harach, H. R., et al. (2002). |  |  |  |  | Yes |  |
| Haraj, N. E., et al. (2016). |  |  |  | Yes |  |  |
| Hodax, J. K., et al. (2016). |  |  |  |  | Yes |  |
| Hut, A., et al. (2019) |  |  |  | Yes |  |  |
| Ikekubo, K., et al. (1989). |  |  |  |  | Yes |  |
| Karagulle, E., et al. (2009). |  |  |  |  |  |  |
| Kitahara, C. M., et al. (2018). |  |  |  |  | Yes |  |
| Kneafsey, B., et al. (1994). |  | Yes |  |  |  |  |
| Lee, E. S., et al. (2013). |  |  |  |  | Yes |  |
| Ly, S., et al. (2016). |  |  |  |  | Yes |  |
| Miccoli, P., et al. (2006). |  |  |  |  | Yes |  |
| Mizukami, Y., et al. (1994). |  |  |  |  | Yes |  |
| Niedziela, M., et al. (2002). |  |  |  |  | Yes |  |
| Pacini, F., et al. (1988). |  |  |  |  | Yes |  |
| Pazaitou-Panayiotou, K., et al. (2008). |  |  |  |  | Yes |  |
| Rosler, H., et al. (1984). |  |  |  |  | Yes |  |
| Rudoni, M., et al. (2010). |  |  | Yes |  |  |  |
| Ruggieri, M., et al. (1999). | Yes |  |  |  |  |  |
| Sahin, M., et al. (2005). | Yes |  |  |  |  |  |
| Shaikh, I. A., et al. (2007). |  |  |  |  | Yes |  |
| Sharma, S. D., et al. (2016) | Yes |  |  |  |  |  |
| Smith, J. J., et al. (2013) |  |  |  |  | Yes |  |
| Smith, M., et al. (1988). |  |  |  |  | Yes |  |
| Sundram, F. X. and P. Mack (1995). | Yes |  |  |  |  |  |
| Tourniaire, J., et al. (1998). |  |  |  | Yes |  |  |
| Trevino, O. G., et al. (1993). |  |  |  | Yes |  |  |
| Zivaljevic, V., et al. (2011). |  |  |  | Yes |  |  |
| Zanella, E., et al. (1998). |  |  |  |  | Yes |  |

**Supplemental Table 4: Incidence of malignancy in hot nodules reported in studies examining only hot nodules.**

| **Authors** | **Incidence of malignancy in AFTN (%)** | **Incidence of malignancy in TMNG (%)** | **Incidence of malignancy in AFTN and TMNG (%)** |
| --- | --- | --- | --- |
| Berker, D., et al. (2011) | 8 | 6 |  |
| Cakir, M., et al. (2007) | 6 | 7 |  |
| Cappelli, C., et al. (2006). | 4 | 4 |  |
| Choong, K. C. and C. R. McHenry (2015). |  | 5 |  |
| Christensen, S. B., et al. (1984). | 0 |  |  |
| Das, A. B., et al. (1996). |  |  | 0 |
| David, E., et al. (1995). | 19 |  |  |
| de Luca, F., et al. (1986). | 13 |  |  |
| Erdogan, M. F., et al. (2003). | 0 | 0 |  |
| Eszlinger, M., et al. (2014). |  |  | 12 |
| Giles, Y., et al. (2008). | 12 | 6 |  |
| Harach, H. R., et al. (2002). |  |  | 7 |
| Hodax, J. K., et al. (2016). | 6 |  |  |
| Karagulle, E., et al. (2009). | 8 | 12 | 10 |
| Kitahara, C. M., et al. (2018). |  |  | 4 |
| Lee, E. S., et al. (2013). |  |  | 26 |
| Ly, S., et al. (2016). | 0 | 0 | 0 |
| Niedziela, M., et al. (2002). |  |  | 29 |
| Miccoli, P., et al. (2006). | 4 | 8 |  |
| Mizukami, Y., et al. (1994). | 12 |  |  |
| Pacini, F., et al. (1988). | 3 | 19 |  |
| Shaikh, I. A., et al. (2007). |  | 26 |  |
| Zanella, E., et al. (1998). | 20 | 2 |  |
| Smith, M., et al. (1988). | 7 |  |  |
| Ikekubo, K., et al. (1989). | 44 |  |  |

Supplemental Figure 1. Pooled odds ratio of combined hot nodules compared with non-toxic nodules excluding pediatric patients and those with known TSHR mutations.


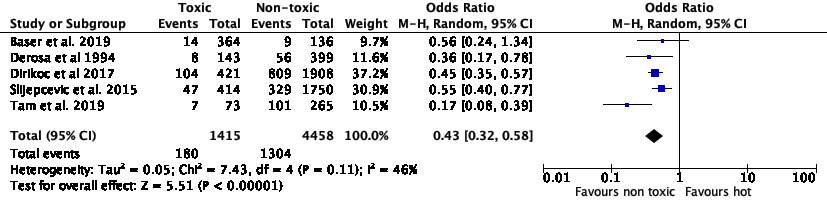


Increased malignancy rate in non-toxic nodules

Increased malignancy rate in hot nodules
